# Tobacco Smoking a Potential Risk Factor in Transmission of COVID-19 Infection

**DOI:** 10.12669/pjms.36.COVID19-S4.2739

**Published:** 2020-05

**Authors:** Naseer Ahmed, Afsheen Maqsood, Tariq Abduljabbar, Fahim Vohra

**Affiliations:** 1 Naseer Ahmed Associate Professor, Department of Prosthodontics, Altamash Institute of Dental Medicine, Karachi 75500, Pakistan; 2 Afsheen Maqsood Assistant Professor, Department of Oral Pathology, Altamash Institute of Dental Medicine, Karachi 75500, Pakistan; 3 Prof. Tariq Abduljabbar Department of Prosthetic Dental Science, College of Dentistry, King Saud University, Riyadh 11545, Saudi Arabia. Research Chair for Biological Research in Dental Health, King Saud University, Riyadh, Saudi Arabia; 4 Prof. Fahim Vohra Department of Prosthetic Dental Science, College of Dentistry, King Saud University, Riyadh 11545, Saudi Arabia. Research Chair for Biological Research in Dental Health, King Saud University, Riyadh, Saudi Arabia

**Keywords:** COVID-19 Infection, Risk Factor, Tobacco Smoking, Viral Transmission

## Abstract

Corona Virus disease 2019 (COVID-19) is a global pandemic and is caused by Severe Acute Respiratory Syndrome Coronavirus-2 (SARS-CoV-2) group of viruses. To date, April 25, 2020, more than 2.4 million humans are infected and more than a hundred thousand deaths have been reported from more than 200 countries from COVID-19. There is no evidence-based treatment for the infection and prevention of transmission using social distancing, isolation and hygiene measures is widely recommended. Tobacco smoking is rampant in communities around the globe and the addiction to tobacco results in deaths of more than 8 million individuals each year. As COVID-19 transmits through salivary droplets and causes severe lung pneumonia, tobacco smokers are also at high risk of severe COVID-19 infection due to poor lung function, cross-infection and susceptible hygiene habits. Smoking tobacco (cigarette, e-cigarettes or waterpipe) produces exhaled smoke, coughing or sneezing, aerosols containing SARS-CoV-2 in the surroundings and contaminating surfaces. Therefore, smoking tobacco is a possible mode of transmission for the virus for both active and passive smokers. Smoking should be considered a risk factor for the disease transmission until further availability of evidence and measures to limit its direct and indirect effects should be implemented within the community.

Corona Virus Disease 2019 (COVID-19) is a global pandemic and is caused by SARS-CoV-2 group of viruses.^1^ The viral disease originated from the Chinese city of Wuhan in December 2019 after confirmation by active testing of patients by Center for Disease Prevention and Control of China and WHO.^1^ It is similar to Severe Acute Respiratory Syndrome Coronavirus-1 (SARS-CoV-1) and Middle East Respiratory Syndrome Coronavirus (MERS-CoV) with its origin in horseshoe bats and pangolins as intermediate hosts.^2^,^3^ To date, more than 2.4 million humans are infected and more than a hundred thousand deaths have been reported from more than 200 countries from COVID-19. The quickly expanding number of cases and documented proof of human-to-human transmission proposes that the virus is more infectious and contagious than SARS-CoV-1 and MERS-CoV.^4^ The virus that causes COVID-19 infects individuals of all age groups. The main source of viral transmission is respiratory droplets, aerosols, salivary contamination, personal contact and contaminated surfaces.^5^ Symptomatic patients transmit the disease predominantly, however asymptomatic patients during the 14-day incubation period are able to transmit the infection.^6^ From earlier reports, the fatality rate of COVID-19 is lower than MERS-CoV and SARS-CoV-1 at 0.39% to 4.05%.^7^ The common symptoms include, fever, body aches, sore throat, cough, headache, diarrhea and shortness of breath.^8^ Around a quarter of the symptomatic patients develop severe symptoms of respiratory distress, shock, renal failure and arrhythmia.^9^ It is believed that patients with existing chronic disease (uncontrolled diabetes, chronic lung disease, coronary heart disease), immune-compromised state and smokers are severely affected.^10^ Treatment for COVID-19 patients include symptomatic (antipyretics, analgesics) and supportive, with no confirmed antiviral therapy.^11^ The risk of severe infection is unpredictable, however older individuals (60 yrs. and above) show a higher mortality rate with COVID-19 infections.^12^

COVID-19 is diagnosed primarily through epidemiological information of travel, contact tracing with infected patients in addition to Reverse transcription polymerase chain reaction (RT-PCR) of nasopharyngeal swabs, saliva and sputum.^12^,^13^ In recent reports, viral strains have been reported up to four weeks of infection.^13^,^14^ And the use of salivary fluid is suggested as a diagnostic tool for early detection of COVID-19 patients.^13^ Saliva in the form of droplets can transmit from COVID-19 infected to healthy individuals during cough, sneeze and conversation in close proximity. In addition, smoking tobacco (cigarette, e-cigarette, waterpipe) involves a frequently contact of saliva with hands and involved devices, which is a possible source of viral spread. Therefore, to prevent transmission, World Health Organization (WHO) has published guidelines which must be practiced consistently.^15^ These include washing of hands for 20 seconds at least with water and soap along with supporting use of alcohol-based sanitizers.^15^ To meticulously practice social distancing by maintaining 1-meter distance between individuals.^15^ Avoid contact with mouth, nose and eyes using hands. It is also recommended to cough and sneeze in elbows followed by hand sanitization.^15^

Tobacco smoking is rampant in communities and the addiction to tobacco and its fashioned use is widespread around the globe. The use of tobacco products kills almost 50% of its users. Tobacco kills in excess of 8 million individuals each year. 5. 7 million mortalities are due to the direct tobacco use, while 1.2 million due to the consequence of non-smokers being presented to tobacco smoke. Worldwide, around 80% (1.1 billion) tobacco smokers live in low income-based countries where malnourishment is also a serious threat to immunity levels.^16^ Tobacco smokers are also at high risk of severe COVID-19 infection due to compromised lung function, cross-infection and susceptible hygiene habits. Smoking predisposes to severe lung diseases by impairing lung substance resulting in diseases like Chronic obstructive pulmonary disease, Emphysema, Chronic Bronchitis and lung cancer. In addition to conventional cigarette smoking, e-cigarettes i.e. Electronic Nicotine Delivery Systems and JUice USB Lighting (ENDS and JUUL), waterpipe and other tobacco smoking devices, use nicotine products. Nicotine chemical derivatives are responsible for eroding the protective lining of lungs, damage of blood vessels and irregular endothelial surface leading to blood clotting and lethal embolism. Therefore, in the presence of existing chronic lung disease, the bronchial status of patients can quickly exacerbate with COVID-19 infection.^17^ Moreover, mortality rate among smokers with COVID-19 infections is 25.8%.^9^ Therefore, strict cross-infection measures are difficult to implement in the presence of smoking. Smoking implies that fingers and contaminated cigarettes shafts are in contact with lips which enhances the chance of transmission of COVID-19 from hand to mouth.^13^,^16^ As a result of smoking tobacco (cigarette, e-cigarettes or waterpipe), due to exhaled smoke, coughing or sneezing, aerosols containing SARS-CoV-2 are produced in abundance in the surroundings, contaminating surfaces. SARS-CoV-2 survives in aerosols and surfaces (plastic, paper and steel) for several hours to days.^16^,^18^ Earlier reports have suggested that SARS-CoV-2 can stay in air as droplets and in micro-aerosols for up to three hours after oral/nasal release.^18^,^19^ In addition, common surfaces used in smoking devices include paper, plastic, metals, on which the virus is found active from four hours up to 2-3 days.^18^ The contaminated surfaces, surrounding air and aerosol due to smoking of tobacco through multiple devices can passively infect non-smokers even if human distancing is practiced. A similar phenomenon of viral infections and transmission has been reported previously, which originated in rodents however had a respiratory and smoking spread.^20^ In Finland, a Puumala Hantavirus outbreak occurred, which was associated to a greater extent with smoking habits apart from other confounding factors.^20^

Continuous global efforts have increased awareness of adverse effects of tobacco smoking and community health care programs at population level, have supported quitting of smoking throughout the years.^21^ However, in the midst of the COVID-19 pandemic, efforts for quitting of smoking tobacco cannot be overemphasized. When isolation and hygiene measures are the most critical management strategies to contain this COVID-19 crisis, quitting and limiting tobacco smoking could be one of the vital measures restricting the viral spread. Therefore, strict smoking etiquettes (Fig.1) need to be adopted by general population and emphasized by the authorities. Smokers should be placed in isolation and smoking only in designated areas with strict hygiene measures (cigarette holders or filters) and good ventilation should be permitted. Smoking should be a solitary habit without the presence of humans in close vicinity. Smoking devices should be single use and no re-use or sharing of devices (conventional cigarette, e-cigarette devices, waterpipe) be practiced. All cigarette stubs and devices and their attachments after single use should be disposed off as contaminated.

**Fig.1:**
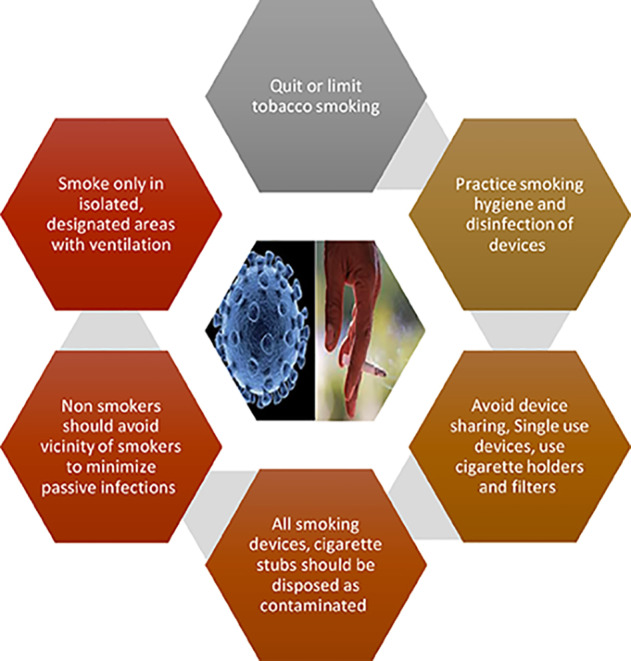
Smoking Etiquettes Amid COVID-19 Outbreak.

## CONCLUSION

In spite of the presence of extraordinary isolation measures, the COVID-19 is in a state of continuous spread in different countries and local territories. Due to its nature of human-to-human transfer, social distancing and strict hygiene protocols are the main strategies in limiting COVID-19 among communities. Tobacco smoking with the use of cigarettes and other devices including electronic cigarettes, Vape, JUUL and water-pipes, threatens the spread of salivary droplets in the form of aerosols specially in cases of SARS-CoV-2 infected asymptomatic smokers. Smoking in groups involves frequent physical approximation and salivary fluid contact that makes it not only a risk factor for COVID-19 but also to other infectious agents acquired from environment through respiration. In addition, transmission of infection due to sharing of some smoking devices including Waterpipe and e-cigarettes can be catastrophic. The occurrence of passive infection through smoke and aerosol from asymptomatic infected smokers is also conceivable. Therefore, smokers should be encouraged to quit tobacco use, especially in public and in groups. In addition, smoking areas must be well ventilated and should be for single individual use. Smokers should use single use devices with strict smoking etiquettes and safe hygiene measures. Government and authorities must provide rigid guidelines applied to public and work places to encourage quitting and banning of smoking. To provide supportive data, future studies reporting smoker specific COVID-19 infection findings related to disease transmission, onset, pattern, progress, management and recovery are recommended.

### Authors’ Contribution

**NA:** Literature review, study design, manuscript writing, final manuscript approval. **AM:** Study design, manuscript drafting, data analysis, manuscript approval. **TA:** Writing, revise, editing and final manuscript approval. **FV:** Literature review, manuscript design and preparation, Data collection, manuscript approval and editing.

## References

[1] Lu H, Stratton CW, Tang YW (2020). Outbreak of pneumonia of unknown etiology in Wuhan China:the mystery and the miracle [published January 16 2020]. J Med Virol.

[2] Chan JF, Yuan S, Kok KH, To KK, Chu H, Yang J (2020). A familial cluster of pneumonia associated with the 2019 novel coronavirus indicating person-to-person transmission:a study of a family cluster. Lancet.

[3] Lu R, Zhao X, Li J, Niu P, Yang B, Wu H (2020). Genomic characterisation and epidemiology of 2019 novel coronavirus:implications for virus origins and receptor binding. Lancet.

[4] Hui DS, Azhar EI, Madani TA, Ntoumi F, Kock R, Dar O (2020). The continuing 2019-nCoV epidemic threat of novel coronaviruses to global health-The latest 2019 novel coronavirus outbreak in Wuhan, China. Int J Infect Dis.

[5] Nair P, Wenzel S, Rabe KF, Bourdin A, Lugogo NL, Kuna P (2017). Oral glucocorticoid–sparing effect of benralizumab in severe asthma. N Eng J Med.

[6] Arabi YM, Murthy S, Webb S COVID-19:a novel coronavirus and a novel challenge for critical care. Intens Care Med.

[7] Centers for Disease Control and Prevention 2020 Disease burden of influenza.

[8] Guan WJ, Ni ZY, Hu Y, Liang WH, Ou CQ, He JX (2020). Clinical characteristics of coronavirus disease 2019 in China. New Eng J Med.

[9] Wang D, Hu B, Hu C, Zhu F, Liu X, Zhang J (2020). Clinical characteristics of 138 hospitalized patients with 2019 novel coronavirus–infected pneumonia in Wuhan, China. JAMA.

[10] Liu K, Fang YY, Deng Y, Liu W, Wang MF, Ma JP (2020). Clinical characteristics of novel coronavirus cases in tertiary hospitals in Hubei Province. Chin Med J.

[11] Tozzi A, D'Amato G Cross-reactivity between COVID-19 and childhood vaccines?Electronic response to:del Rio C;Malani PN 2020. 2019 Novel Coronavirus-Important Information for Clinicians. JAMA.

[12] Huang C, Wang Y, Li X, Ren L, Zhao J, Hu Y (2020). Clinical features of patients infected with 2019 novel coronavirus in Wuhan, China. Lancet.

[13] Sabino-Silva R, Jardim AC, Siqueira WL Coronavirus COVID-19 impacts to dentistry and potential salivary diagnosis. Clin Oral Investigations.

[14] Zuanazzi D, Arts EJ, Jorge PK, Mulyar Y, Gibson R, Xiao Y (2017). Postnatal identification of Zika virus peptides from saliva. J Den Res.

[15] World Health Organization. Clinical management of severe acute respiratory infection when novel coronavirus (nCoV) infection is suspected:Interim Guidance, 25 January 2020 (2020). World Health Organization.

[16] Sherman CB (1991). Health effects of cigarette smoking. Clin Chest Med.

[17] Palipudi K, Rizwan SA, Sinha DN, Andes LJ, Amarchand R, Krishnan A (2014). Prevalence and sociodemographic determinants of tobacco use in four countries of the World Health Organization:South-East Asia region:findings from the Global Adult Tobacco Survey. Indian J Cancer.

[18] Benjamin RM (2011). Exposure to tobacco smoke causes immediate damage:A report of the Surgeon General. Public Health Reports.

[19] Zhu N, Zhang D, Wang W, Li X, Yang B, Song J (2020). A novel coronavirus from patients with pneumonia in China 2019. N Eng J Med.

[20] Vapalahti K, Virtala AM, Vaheri A, Vapalahti O (2010). Case-control study on Puumala virus infection:smoking is a risk factor. Epidemiol Infect.

[21] Jha P, Peto R (2014). Global effects of smoking, of quitting, and of taxing tobacco. N Engl J Med.

